# A *Francisella tularensis* Live Vaccine Strain That Improves Stimulation of Antigen-Presenting Cells Does Not Enhance Vaccine Efficacy

**DOI:** 10.1371/journal.pone.0031172

**Published:** 2012-02-15

**Authors:** Deanna M. Schmitt, Dawn M. O'Dee, Joseph Horzempa, Paul E. Carlson, Brian C. Russo, Jacqueline M. Bales, Matthew J. Brown, Gerard J. Nau

**Affiliations:** 1 Department of Microbiology and Molecular Genetics, University of Pittsburgh School of Medicine, Pittsburgh, Pennsylvania, United States of America; 2 Department of Natural Sciences and Mathematics, West Liberty University, West Liberty, West Virginia, United States of America; 3 Department of Medicine – Division of Infectious Diseases, University of Pittsburgh School of Medicine, Pittsburgh, Pennsylvania, United States of America; 4 Center for Vaccine Research, University of Pittsburgh School of Medicine, Pittsburgh, Pennsylvania, United States of America; Institut de Pharmacologie et de Biologie Structurale, France

## Abstract

Vaccination is a proven strategy to mitigate morbidity and mortality of infectious diseases. The methodology of identifying and testing new vaccine candidates could be improved with rational design and *in vitro* testing prior to animal experimentation. The tularemia vaccine, *Francisella tularensis* live vaccine strain (LVS), does not elicit complete protection against lethal challenge with a virulent type A *Francisella* strain. One factor that may contribute to this poor performance is limited stimulation of antigen-presenting cells. In this study, we examined whether the interaction of genetically modified LVS strains with human antigen-presenting cells correlated with effectiveness as tularemia vaccine candidates. Human dendritic cells infected with wild-type LVS secrete low levels of proinflammatory cytokines, fail to upregulate costimulatory molecules, and activate human T cells poorly *in vitro*. One LVS mutant, strain 13B47, stimulated higher levels of proinflammatory cytokines from dendritic cells and macrophages and increased costimulatory molecule expression on dendritic cells compared to wild type. Additionally, 13B47-infected dendritic cells activated T cells more efficiently than LVS-infected cells. A deletion allele of the same gene in LVS displayed similar *in vitro* characteristics, but vaccination with this strain did not improve survival after challenge with a virulent *Francisella* strain. *In vivo*, this mutant was attenuated for growth and did not stimulate T cell responses in the lung comparable to wild type. Therefore, stimulation of antigen-presenting cells *in vitro* was improved by genetic modification of LVS, but did not correlate with efficacy against challenge *in vivo* within this model system.

## Introduction

The development of vaccines is essential to combat harmful infectious diseases [1]. Obtaining licensure after discovery of a vaccine, however, can take up to 20 years due to the stringent testing required to confirm the safety and efficacy of the vaccine [2]. To expedite this process, *in vitro* tests could be developed to define correlates of protection and identify more promising vaccine candidates. These assays would be particularly beneficial with vaccine candidates for highly pathogenic organisms, such as the bacterium *Francisella tularensis*, when challenge studies cannot be performed in humans because of contemporary regulations that govern clinical trials [3].

*F. tularensis* is the causative agent of tularemia [4]. This zoonotic disease is endemic in North America and parts of Europe and Asia, and outbreaks in these regions are frequently associated with the handling of infected animals or transmission by arthropod vectors [4], [5]. *F. tularensis* is also classified by the Centers for Disease Control and Prevention as a Category A bioterrorism agent [6]. When inhaled, less than 10 organisms can cause an acute pneumonia that is lethal in up to 60% of infected individuals if left untreated [7]. The World Health Organization predicted that if virulent *F. tularensis* was aerosolized over a metropolitan area of five million people, more than 19,000 people would die and 250,000 individuals would be incapacitated [6]. An effective vaccine would be useful to reduce the number of naturally occurring tularemia cases and to protect against a possible intentional release.

To date, two different types of tularemia vaccines have been studied in humans. The Foshay vaccine consisted of chemically killed *F. tularensis* and was effective at reducing the incidence of laboratory-acquired tularemia cases from approximately 100% to 30% in the 1950s [8], [9]. However, killed *F. tularensis* provided only minimal protection from aerosol type A *Francisella* challenge in a vaccine trial [10]. Researchers in the former Soviet Union took a different approach and developed a range of live attenuated *Francisella* strains to immunize people against tularemia [11]. One of these strains, a live attenuated strain of *F. tularensis* subsp. *holarctica*, live vaccine strain (LVS), was superior to the Foshay-type vaccines at providing protection [10], [12], [13]. While two clinical studies involving small numbers of human vaccinees demonstrated effectiveness of LVS against aerosol challenge by virulent type A *Francisella* [10], [12], a later study showed variable efficacy that diminished over time [14]. Vaccination of individuals by aerosol improved the efficacy of LVS but this required a high dose of 10^6^ to 10^8^ organisms which frequently resulted in severe adverse side effects [14].

Currently, LVS is not approved by the Food and Drug Administration (FDA) due to concerns about its undefined attenuation, mechanism of protection, and reversion frequency [15]. In order to obtain FDA approval, several groups are working to address these issues. Recent work by Salomonsson *et al.* identified two regions of difference, RD18 and RD19, which are deleted in LVS and account for its attenuation [16]. Additional studies improved the manufacturing process of LVS in compliance with good manufacturing practice guidelines [17]. This new lot of LVS was further characterized in human phase I clinical trials [18]. Researchers are also introducing mutations into LVS in order to improve its efficacy and bolster attenuation. One example is an LVS mutant deficient in iron superoxide dismutase (*sodB_Ft_*). Compared to LVS, *sodB_Ft_* increases median time to death and percent survival of C57BL/6 mice from pulmonary type A *Francisella* challenge [19]. As work toward the licensing of LVS continues, attempts have been made to replace LVS with a genetically defined, attenuated type A *Francisella* strain. For example, Schu S4 ΔFTT_1103 and Schu S4 Δ*clpB*, provide 75% and 60% protection, respectively, from virulent type A *Francisella* challenge in BALB/c mice [20], [21]. Nevertheless, LVS remains the leading tularemia vaccine to date that has shown activity in humans [10], [12].

A potential limitation of LVS as a vaccine is its relative stimulation of antigen-presenting cells (APCs). Published work has shown LVS stimulates murine and human DCs [22], [23], though it is now known that culture conditions influence stimulation of innate immunity [24], [25], [26], [27], [28]. In contrast, other studies have shown that LVS suppresses the activation of murine macrophages [24], [29], [30], [31], [32] and dendritic cells (DCs) [32]. Murine macrophages and DCs cultured with LVS produce little to no proinflammatory cytokines *in vitro* compared to DCs cultured with other bacteria or TLR ligands [24], [29], [30], [32]. Stimulation with TLR ligands such as *Escherichia coli* LPS fails to restore cytokine secretion by these cells suggesting that LVS is actively suppressing TLR signaling [24], [25], [29], [32]. Another study showed that this suppression is due to downregulation of critical inflammatory signaling pathways involved in MAPK and NF-κB activation [30].

In this study, we tested whether *in vitro* screening of potential tularemia vaccine candidates for enhanced stimulation of APCs would improve a candidate's immunogenicity, and ultimately protection after challenge. After initial testing of several LVS strains, we evaluated one genetic locus in detail with mutant strains that showed desirable vaccine characteristics *in vitro*, including attenuation in macrophages and enhanced DC stimulation. Despite these traits, they did not predict better protection against virulent type A *Francisella* challenge.

## Materials and Methods

### Ethics Statement

Human cells were purified from discarded buffy coats obtained from the Central Blood Bank (Pittsburgh, PA). The use of these samples was reviewed and approved by the Institutional Review Board of the University of Pittsburgh, which made a “no human subjects” determination and waived requirement for consent. All research involving animals was conducted in accordance with animal care and use guidelines, and animal protocols were approved by the University of Pittsburgh Animal Care and Use Committee (protocols 1003587 and 1002514).

### *Francisella* strains and growth conditions

For cultivation of *F. tularensis* LVS strains and Schu S4, frozen stock cultures were streaked onto chocolate II agar plates and incubated at 37°C, 5% CO_2_ for 2–3 days. LVS strains were grown in Chamberlain's chemically defined broth medium (CDM) [33] or MH broth [Mueller-Hinton broth (Difco) supplemented with 0.1% glucose, 0.025% ferric pyrophosphate (Sigma), and IsoVitaleX (Becton Dickinson)] for *in vitro* infections. For mouse vaccinations, MH broth or TSBc [trypticase soy broth (BD Biosciences) supplemented with 0.1% L-cysteine hydrochloride monohydrate (Fisher)] was used for culturing of LVS strains. Schu S4 was grown in MH broth for infections of vaccinated mice. Broth cultures were grown at 37°C with shaking for 14–18 hours. When required, antibiotics were added to the media at the following concentrations: kanamycin at 10 µg/ml, chloramphenicol at 5 µg/ml, and hygromycin at 200 µg/ml.

### Generation of formalin-fixed *Francisella tularensis* Schu S4 (ffSchu S4)

Schu S4 was grown in MH broth as described above. Following overnight culture, bacteria were washed, resuspended in PBS (Gibco), and adjusted to an OD_600_ of 0.3. Bacteria were then resuspended in 10% buffered formalin (Fisher) and incubated at 25°C for 10 min with shaking (200 rpm). Bacteria were washed five times and resuspended in PBS for an approximate concentration of 1–3×10^8^ CFU/ml. Bacterial killing was confirmed by plating of formalin-fixed Schu S4 on chocolate II agar plates in which no colonies were observed following extensive incubation (data not shown). Prior to formalin fixation, an aliquot of the bacterial suspension was removed and tested for viable CFU by plating serial dilutions on chocolate II agar.

### Construction of LVS mutants

Construction of LVS strain 1664d was described previously [34]. The *F. tularensis* LVS ΔcapC mutant was generated using homologous recombination with a suicide plasmid. This plasmid contained two segments homologous to regions flanking FTL_1415 and one third of the 5′ and 3′ ends of this ORF, surrounding a chloramphenicol acetyltransferase gene (*cat*) under the control of the *F. tularensis groE* promoter (Table 1). Linearized plasmid was electroporated into LVS [35] and double cross-over events were selected on cysteine heart agar with 5% defibrillated rabbit blood containing 2.5 µg/ml chloramphenicol. Recombination was confirmed by PCR (data not shown).

**Table 1:** Bacterial strains, plasmids, and primers used in this study.

Strain, plasmid, or primer	Description	Source or Reference
***F. tularensis*** ** Strains**		
LVS	*F. tularensis* subsp. *holarctica* live vaccine strain	Karen Elkins
13B47	LVS with the Tn from pSD26 disrupting FTL_0883 in base pair 115 of 842 from the 5′ end	This study
1664d	LVS *deoB* (FTL_1664) disruption mutant	[25]
ΔcapC	LVS containing *cat* replacing the central one third of *capC* (FTL_1415)	This study
ΔFTL_0883	LVS FTL_0883 in-frame deletion mutant	[38]
ΔFTL_0883::pJH1-FTL-0883	LVS ΔFTL_0883 cis-complement	[38]
***E. coli*** ** Strains**		
sd-4	Streptomycin-dependent *E. coli* mutant (ATCC 11143)	ATCC 11143
**Plasmids**		
pSD26	*E. coli* shuttle plasmid (colE1, Ap^R^) encoding the C9 transposase and *himar* transposon (Km^R^)	Simon Dillon and Eric Rubin
**Primers**		
capC-1	5′–CCGCGGAAGCGACACATGGACTTTTGA–3′	This study
capC-2	5′–GAATTCAATATGATAATAGTTACTATAACT–3′	This study
capC-3	5′–ATGCATTTATATTATCCCTGGACTTAT–3′	This study
capC-4	5′–ACTAGTTTAGATTTTTTATTATCGTTA–3′	This study

To generate strain 13B47, plasmid pSD26 (a gift from Eric Rubin and Simon Dillon) was electroporated into LVS as previously described [36]. pSD26 is an *E. coli* plasmid delivery vector (*colE1*, Ap^R^) that encodes a *Himar1* transposase [37] and a transposon containing a kanamycin resistance cassette under the control of the *F. tularensis groE* promoter (Table 1). Following recovery in trypticase soy broth supplemented with 0.1% cysteine, bacteria were plated on cysteine heart broth with 5% defibrinated rabbit blood containing kanamycin (5 µg/ml). Colonies that emerged in the presence of kanamycin were isolated and screened for lack of response to extracellular spermine [38]. The selection phenotype was lack of growth in CDM plus an inhibitor of endogenous polyamine biosynthesis, dicyclohexylamine [38]. Transposon mutants that failed to grow were then tested for their ability to stimulate cytokines [38]. Strain 13B47 elicited high concentrations of TNF-α from human macrophages and had a transposon insertion in FTL_0883 [38]. DNA sequencing showed that the precise location of this transposon was in base pair 115 of 842 from the 5′ end of FTL_0883. Construction of an in-frame deletion mutant, ΔFTL_0883, and a cis-complement strain was described previously [38].

### Infection of macrophages and DCs with *F. tularensis* LVS strains

Human monocytes were differentiated into macrophages and DCs by *in vitro* culture as described previously [34]. For generation of murine DCs, bone marrow was flushed from femurs and tibias of C57BL/6J mice with complete DC medium [DMEM supplemented with 10% heat-inactivated FBS, 25 mM HEPES, 1% non-essential amino acids, 1% sodium pyruvate, 1% GlutaMAX and 0.1% 2-mercaptoethanol (all from Gibco)]. Freshly isolated cells were washed and red blood cells were lysed with ACK Lysis Buffer (Gibco). After washing and counting, cells were resuspended in complete DC media supplemented with 500 U/ml GM-CSF (eBioscience) and seeded into T75 flasks or 24-well plates at a concentration of 20–30 million cells per flask or 1×10^6^ cells/well, respectively. Cells were incubated at 37°C, 5% CO_2_ for 5–6 days and fresh complete DC media with 500 U/ml GM-CSF was added every 2 days. Bone marrow DCs (BMDCs) were purified using CD11c magnetic beads (Miltenyi Biotec) per the manufacturer's instructions. The resulting cells were greater than 90% CD11c^+^ as assessed by flow cytometry.

For cytokine and flow cytometry experiments, human cells were washed and resuspended in DMEM supplemented with 1% human serum, 25 mM HEPES, and 1% GlutaMAX. Murine BMDCs were washed and resuspended in DMEM supplemented with 10% FBS, 25 mM HEPES, 1% non-essential amino acids, 1% sodium pyruvate, 1% GlutaMAX and 0.1% 2-mercaptoethanol (all from Gibco). DCs and macrophages were seeded into 24-well plates (Costar) at 5×10^5^ cells/well and 1.5×10^5^ cells/well, respectively. Infections were conducted using two different methods with the goal of maintaining eukaryotic cell viability. A multiplicity of infection (MOI) of 10 was used for 24-hour co-cultures at 37°C with 5% CO_2_, allowing cultures to proceed without washing. In other experiments, DCs were cultured with bacteria at an MOI of 500 for two hours as described below, which results in a high infection rate but with minimal effects on cell viability [25], [34]. As a positive control, macrophages and DCs were stimulated with *E. coli* strain sd-4 (ATCC 11143) [39]. Supernatants were collected at various times post infection (6, 12, 24, and 48 hours) and DCs were prepared for flow cytometric analysis.

Gentamicin protection assays were used to assess intracellular growth [40]. Here, macrophages and DCs were seeded in Primaria 96-well culture dishes (BD Biosciences) at a density of 5×10^4^ cells/well and infected with bacteria at an MOI of 500. After two hours, cells were incubated with Hanks balanced salt solution (Gibco) containing gentamicin (20 µg/ml) for 20 min to kill extracellular bacteria. Cultures were then washed three times with warm Hank's balanced salt solution and incubated at 37°C with 5% CO_2_ for another 22 h with fresh culture medium. Actual MOIs were measured by plating serial dilutions of inocula on chocolate II agar plates. At the indicated time points post infection, viable CFU were measured as described previously [34], [41]. Bacterial growth was compared using Student's *t*-test.

For DC-T cell co-culture assays, DCs were resuspended in complete T cell medium [DMEM supplemented with 10% heat-inactivated FBS, 25 mM HEPES, 1% non-essential amino acids, 1% sodium pyruvate, 1% GlutaMAX and 0.1% 2-mercaptoethanol (all from Gibco)] and seeded in 96-well round bottom plates (BD Biosciences) at a density of 2×10^4^ cells/well. DCs were cultured with different *F. tularensis* LVS strains at an MOI of 10 for 24 hours prior to co-culture with T cells (see “Human DC-CD4^+^ T cell co-culture”).

### Flow cytometry and analysis of human monocyte-derived DCs

Surface markers on *F. tularensis*-infected human monocyte-derived DCs were evaluated by flow cytometric analysis. Following infection, DCs were removed from 24-well plates using a 2 mM EDTA solution. Cells were washed once and resuspended in FACS staining buffer [0.1% bovine serum albumin and 0.1% sodium azide in PBS]. Nonspecific antibody binding was blocked with human FcR Blocking Reagent (Miltenyi Biotec). Cells were stained with fluorescein isothiocyanate (FITC)-conjugated anti-CD1b (clone MT-101, AbD Serotec), phycoerythrin (PE)-conjugated anti-CD86 (clone IT2.2, eBioscience), PE-Cy5-conjugated anti-CD80 (clone 2D10.4, eBioscience), and PE-Cy7-conjugated anti-HLA-DR (clone LN3, eBioscience) at 4°C for 30 min. Isotype control antibodies were included in each experiment to confirm specificity of staining. After washing and fixing in 2% paraformaldehyde for 30 min at 4°C, cells were analyzed using a LSRII flow cytometer (BD Biosciences) and FlowJo Software (Tree Star). Statistically significant differences in CD80, CD86, and HLA-DR expression by infected DCs were determined by one-way ANOVA, followed by Bonferroni comparison of means.

### Human DC-CD4^+^ T cell co-culture

DC-T cell co-cultures were performed similarly to previous studies [42], [43], [44]. CD4^+^ T cells were purified from human peripheral blood mononuclear cells that passed through the Optiprep gradient by positive selection using the Dynal CD4 Positive Isolation Kit (Invitrogen) per the manufacturer's instructions. These cells were >95% CD3^+^CD4^+^ T cells as assessed by flow cytometry. Purified CD4^+^ T cells were then stained with 2.5 µM CFSE for 10 min at 37°C, washed, and resuspended in complete T cell medium. CFSE-labeled T cells from a single donor were then added to DCs from a different donor that had been exposed to bacteria. DC-T cell co-cultures were performed in a 96-well round bottom plate at a ratio of 10∶1 (2×10^5^ T cells/2×10^4^ DCs/well) for a period of 5 days at 37°C with 5% CO_2_. After harvesting supernatants, cells were washed once and resuspended in FACS staining buffer, treated with human FcR Blocking Reagent (Miltenyi Biotec), and stained with APC-conjugated anti-CD4 (clone OKT4, eBioscience) at 4°C for 30 min. After washing and fixing in 2% paraformaldehyde for 30 min at 4°C, fluorescence was measured using a FACSCalibur flow cytometer (BD Biosciences) and analyzed with FlowJo Software (Tree Star). For T cell proliferation, CFSE^low^ cells were measured in the CD4^+^ gate. Statistically significant differences in the percentage of proliferating T cells following co-culture with infected DCs were determined by one-way ANOVA, followed by Bonferroni comparison of means.

### Mice

Six- to eight-week old female C57BL/6J mice were purchased from Jackson Laboratories (Bar Harbor, ME). Mice were housed in microisolator cages under specific pathogen-free conditions in a biosafety level-3 animal facility.

### Immunization of mice

LVS and ΔFTL_0883 were cultured in MH broth or TSBc as described above. Mice were immunized subcutaneously (s.c.) or intratracheally (i.t.). Vaccinations were performed i.t. by oropharyngeal instillation as described previously [41]. A subset of mice was sacrificed at 2 hours post infection, and their lungs were homogenized and plated to confirm delivery of bacteria to the respiratory tract. Actual administered doses were determined by plating serial dilutions of the inocula onto chocolate II agar plates.

### Infection of mice with *F. tularensis* Schu S4

Schu S4 was grown in MH broth as described above. Mice were infected i.t. with 100 CFU of Schu S4 six weeks following LVS or ΔFTL_0883 vaccination. The actual dose was calculated by plating serial dilutions of the inoculum onto chocolate II agar plates. Following infection, mice were monitored daily for survival.

### Measurements of bacterial burden *in vivo*

Bacterial burdens in the organs of mice vaccinated with LVS strains were measured as previously described [38], [41]. Mice were sacrificed at the indicated time points and lungs, spleens, and livers were removed and homogenized in 1 ml (lungs, spleens) or 2 ml (livers) of TSBc. A portion of the organ homogenates were serially diluted and plated onto chocolate II agar plates. Plates were incubated at 37°C at 5% CO_2_ and individual colonies were enumerated.

### *In vitro* stimulation of lung cells from vaccinated mice

Six weeks following vaccination with LVS or ΔFTL_0883, lungs were excised, minced, and incubated in RPMI (Gibco) supplemented with 12 mg type I collagenase (Gibco), 100 µg DNase I (USB), and 3 mM CaCl_2_ for 30 min at 37°C with shaking (170 rpm). The digested tissue was passed through a 40 µm cell strainer (BD Biosciences) to generate single cell suspensions. Erythrocytes were lysed with ACK Lysis Buffer (Gibco) and remaining cells were washed with RPMI. Viable cells were counted using trypan blue exclusion. Cells were resuspended in complete RPMI [RPMI supplemented with 10% heat-inactivated FBS, 25 mM HEPES, 1% non-essential amino acids, 1% sodium pyruvate, 1% GlutaMAX and 0.1% 2-mercaptoethanol (all from Gibco)] and seeded into 96-well round bottom plates at 1.5×10^6^ cells/well. Lung cells from naïve mice served as controls. BMDCs were generated as described above without CD11c magnetic bead purification. BMDCs were resuspended in complete RPMI and added at a 1∶10 ratio (1.5×10^5^ BMDCs/1.5×10^6^ lung cells) to lung cells.

Preliminary experiments were performed with lung cells from LVS-vaccinated mice to determine the optimal antigen concentration and length of co-culture for this assay. Peak cytokine production was detected after 48 hours of co-culture with similar results observed at 72 hours (data not shown). Little to no cytokine production was detected from lung cells cultured with BMDCs and ffSchu S4 below an MOI of 10 (data not shown). As a result, cells were incubated at 37°C with either media alone or ffSchu S4 at dose of 10 CFU per cell. After 48 hour co-culture, supernatants were collected for analysis of cytokines and chemokines.

### Cytokine and Chemokine Assays

DCs and macrophage supernatants were tested by ELISA using commercially available kits to measure TNF-α (R&D Systems), IL-12p40 (human, R&D Systems; mouse, eBiosciences), and IL-6 (human, R&D Systems) according to the manufacturer's instructions. IFN-γ in supernatants from human DC-CD4^+^ T cell co-cultures was also measured by ELISA (human, R&D Systems). The limits of detection for the ELISAs were: human TNF-α – 15.6 pg/ml, human and mouse IL-12p40 – 31.2 pg/ml, human IL-6 – 9.7 pg/ml, and human IFN-γ – 15.6 pg/ml. Cytokine and chemokine levels in lung supernatants from *in vitro* re-stimulation assays were determined by ELISA (mouse IFN-γ, R&D Systems; mouse IL-17A, Biolegend) or by using the Milliplex 32-plex Mouse Cytokine/Chemokine Panel (Millipore) on a Bio-Plex 200 system (Bio-Rad Laboratories). Analyte concentrations were calculated against the standards using Milliplex Analyst software (version 3.5; Millipore). The limits of detection for the ELISAs were 31.2 pg/ml for mouse IFN-γ and 15.6 pg/ml for mouse IL-17A. Statistically significant differences in cytokine production were identified by one- or two-way ANOVA followed by Bonferroni comparison of means.

## Results

### Limited inflammatory response of human DCs to LVS

**Figure 1:**
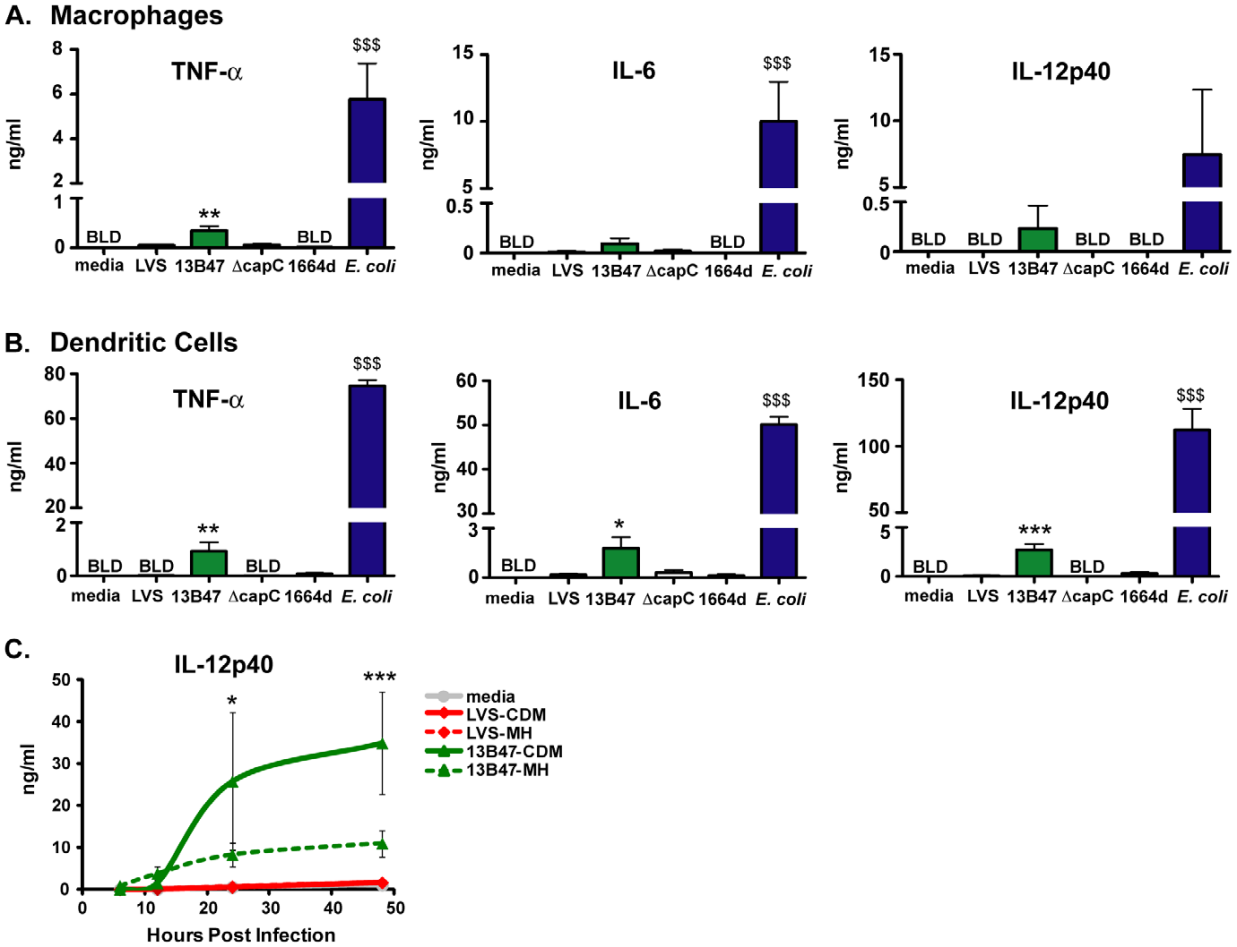
*F. tularensis* LVS strain 13B47 stimulates human monocyte-derived DCs and macrophages to produce proinflammatory cytokines. LVS and LVS mutants, 13B47, ΔcapC, and 1664d, were cultured overnight in a chemically defined media (CDM) or Mueller-Hinton (MH) broth. The four bacterial cultures were used to inoculate macrophages (A, 1.5×10^5^ cells/well) and DCs (B and C, 5×10^5^ cells/well) at an MOI of 10. As a positive control, DCs and macrophages were cultured with *E. coli* strain sd-4 (MOI = 10). Supernatants were harvested after 24 hours (A–B) or at indicated times (C), and TNF-α, IL-6, and IL-12p40 were measured by ELISA. Data are expressed as the mean ± SEM of three individual experiments with different donors. The level of cytokine production from each group was compared by a one (A–B) or two-way ANOVA (C), followed by the Bonferroni comparison of means. ($$$, *p*<0.001 for *E. coli* vs. all other groups). When comparing only the DCs infected with the *F. tularensis* strains, 13B47 elicited higher cytokine production than the uninfected group (A–B) or LVS cultured in the same media (C). *, p<0.05; **, p<0.01; ***, p<0.001. BLD = below limits of detection of the ELISA.

We have shown previously that human macrophages have a limited capacity to produce proinflammatory cytokines following infection with LVS [25]. We hypothesized that human DCs would also be hyporesponsive to LVS stimulation. To test this, human macrophages and DCs were co-cultured with LVS, and then supernatants were harvested and analyzed for the proinflammatory cytokines TNF-α, IL-6, and IL-12p40. Similar to our findings with macrophages (Fig. 1A), LVS elicited little to no proinflammatory cytokines from human DCs (Fig. 1B). Pre-treating LVS with 100% human serum failed to enhance cytokine production (data not shown). As a positive control, human DCs were stimulated with *E. coli* [39]. *E. coli*-stimulated DCs produced significantly higher levels of all cytokines measured compared to untreated DCs or DCs cultured with LVS (Fig. 1B).

### Identification of an immunostimulatory *F. tularensis* LVS strain

We hypothesized that a LVS mutant inducing a stronger proinflammatory response from APCs *in vitro* would be a more effective tularemia vaccine candidate. Surveying pre-existing LVS mutants generated in our laboratory, we tested several for their ability to stimulate cytokine production from human DCs and macrophages (Table 1). Human DCs and macrophages were co-cultured with each of the mutants in parallel with wild-type LVS, and supernatants were analyzed for cytokines. Cytokine production by DCs and macrophages infected with either ΔcapC or 1664d was similar to LVS-infected cells at 24 hours post infection (Fig. 1A–B). In contrast, DCs and macrophages infected with the 13B47 strain produced elevated levels of all cytokines measured (Fig. 1A–B). Similar results were observed when cytokine levels were measured 48 hours after infection (data not shown). Although the cytokine levels elicited by 13B47 were lower than those produced by cells stimulated with *E. coli* (Fig. 1A–B), each was readily detected. Among the LVS strains tested, therefore, 13B47 stimulated the most proinflammatory cytokines from human APCs.

We next assessed whether the medium used to grow the bacteria would influence stimulation of DCs. LVS grown in media containing high levels of polyamines such as CDM stimulates low levels of proinflammatory cytokines from macrophages [25], [27], [38]. To address the effect culture conditions may have on the DC phenotypes observed here, LVS and 13B47 were cultured in CDM or MH broth prior to co-culture with human DCs. At various time points post infection, supernatants were harvested and analyzed for detection of IL-12p40. At 24 and 48 hours post infection, greater than 10-fold higher levels of IL-12p40 were produced by human DCs cultured with 13B47 compared to wild-type LVS (Fig. 1C). IL-12p40 production by DCs was higher regardless of whether 13B47 was cultured in CDM or MH broth (Fig. 1C). This result indicated that induction of cytokine production by 13B47 was not dependent on the growth medium used to culture this strain.

### Maturation of DCs infected with *F.tularensis* strain 13B47

**Figure 2:**
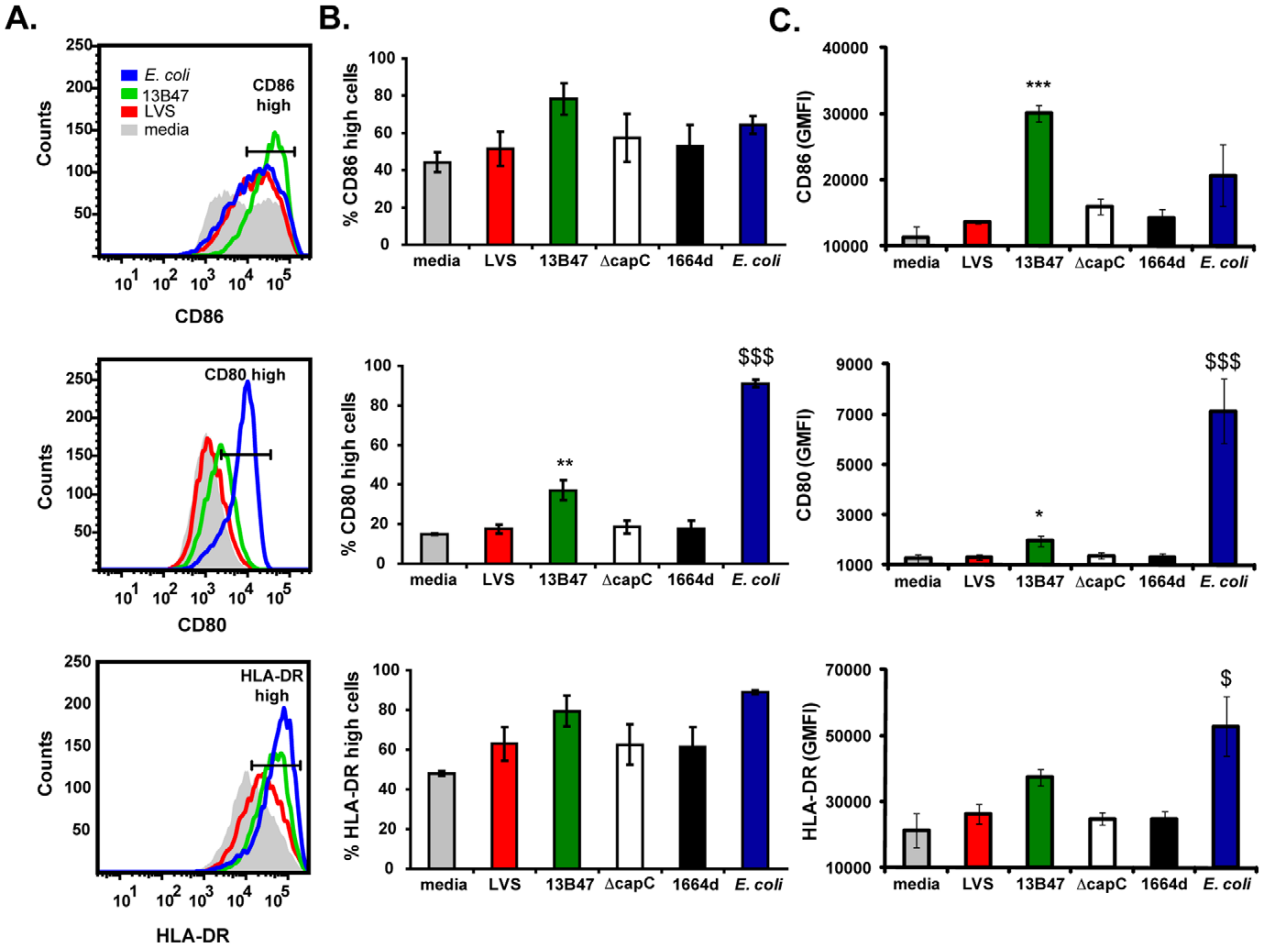
Human monocyte-derived DCs mature following exposure to LVS strain 13B47. DCs were stimulated with either LVS, 13B47, ΔcapC, 1664d, or *E. coli* for 24 hours (MOI = 10). Cells were harvested and analyzed for changes in surface expression of CD86, CD80, and HLA-DR. Cells were gated on CD1b-positive population. (A) Representative histograms for CD86, CD80, and HLA-DR expression on LVS-, 13B47-, and *E. coli*-treated DCs from one experiment. Histograms for ΔcapC- and 1664d-infected DCs were similar to LVS (data not shown). (B) Mean percentages of DCs with high CD86, CD80, and HLA-DR expression (± SEM) from three individual experiments with different donors. (C) Geometric mean fluorescence intensities (GMFI) of CD86, CD80, and HLA-DR (± SEM) on DCs from three individual experiments with different donors. Statistically significant differences in CD86, CD80, and HLA-DR expression by infected DCs were determined by one-way ANOVA, followed by Bonferroni comparison of means (*, p<0.05; **, p<0.01; ***, p<0.001).

In addition to the secretion of cytokines, DCs must undergo a process called maturation in order to efficiently prime T cells and initiate the adaptive immune response [45]. Among these alterations, the expression of MHC and costimulatory molecules increases. Since 13B47 stimulated cytokine production from human DCs, we next evaluated whether these cells also changed their surface phenotype in response to this mutant. The expression of CD80, CD86, and HLA-DR was measured on DCs following culture with either wild-type *F. tularensis* LVS, 13B47, ΔcapC, 1664d, or *E. coli* as a positive control for maturation. LVS elicited little to no change in expression of maturation markers on the surface of human DCs (Fig. 2A–C). Similar results were observed with the LVS mutants ΔcapC and 1664d (Fig. 2B, C). In contrast, the percentage of high-expressing cells and/or geometric mean fluorescence intensity increased after culture with 13B47 for CD80 and CD86 (Fig. 2). A similar trend of heightened expression of HLA-DR was also observed with 13B47-infected DCs (Fig. 2). Likewise, *E. coli*-stimulated DCs increased expression of costimulatory molecules and MHC (Fig. 2). These data suggest that DCs undergo maturation after exposure to *F. tularensis* strain 13B47 and, therefore, may be better suited to initiate an adaptive immune response.

### Growth of 13B47 in human macrophages and DCs

**Figure 3:**
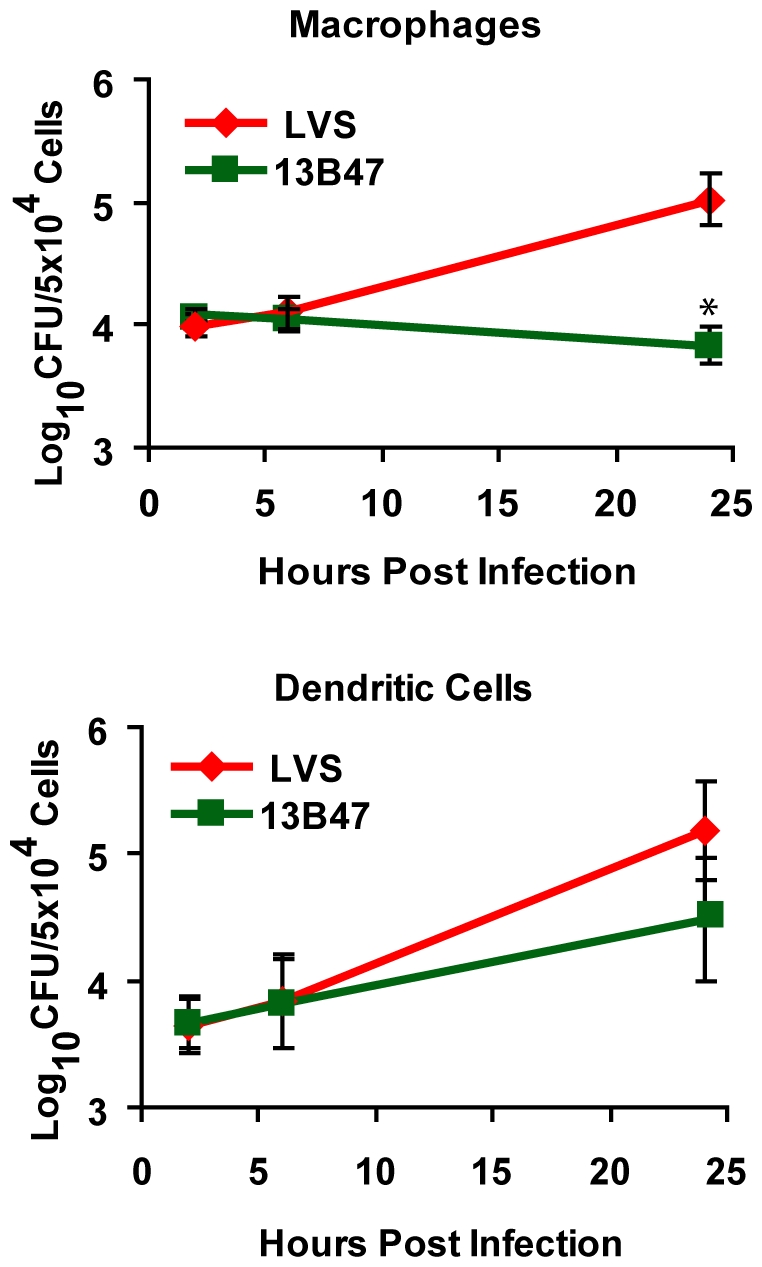
LVS strain 13B47 is attenuated for growth in human macrophages and replicates slowly in DCs. DCs or macrophages were infected in gentamicin protection assays (MOI = 500) with LVS or 13B47 and lysed at the indicated times post infection. Data shown are mean ± SEM from three individual experiments with different donors. Statistically significant differences in growth at 24 hours post infection were determined by Student's *t*-test (*, p<0.05).

Intracellular growth is a hallmark of pathogenic *Francisella* strains. Although 13B47 stimulated APCs to secrete cytokines and upregulate costimulatory molecules, it was unclear if its intracellular growth was altered. To test this, human DCs and macrophages were infected with either wild-type LVS or strain 13B47, and lysed at various times post infection to enumerate intracellular bacteria. 13B47 was attenuated for growth at 24 hours post infection in human macrophages (Fig. 3). Surprisingly, 13B47 was still capable of replicating in human DCs (Fig. 3), albeit with a slightly slower rate compared to wild-type LVS (estimated generation time of 783 minutes versus 275 minutes for wild-type). These phenotypes could not be attributed to a general growth defect since 13B47 grew similar to wild-type LVS in bacterial growth medium (data not shown). These data suggest that, while the cytokine response to 13B47 is similar between macrophages and DCs, these cells differ in their ability to control growth of this mutant.

### Enhanced activation of CD4^+^ T cells by *F. tularensis* strain 13B47-infected DCs

**Figure 4:**
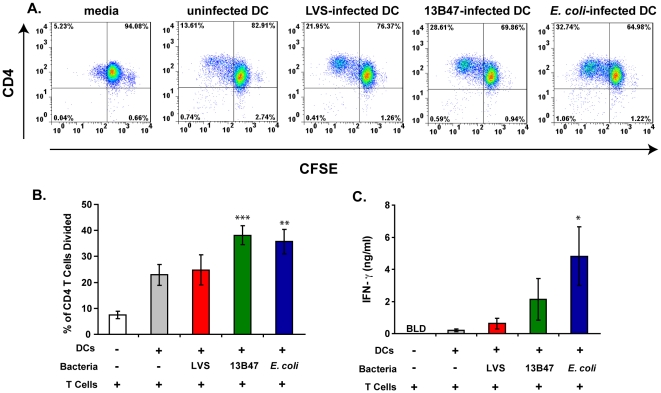
Enhanced proliferation and IFN-γ production by CD4^+^ T cells stimulated with LVS strain 13B47-infected DCs. Purified CFSE-labeled CD4^+^ T cells from a single donor were co-cultured with either *E. coli*-infected, *F. tularensis* LVS-infected, or 13B47-infected DCs from a different donor at a ratio of 10∶1 (2×10^5^ T cells/2×10^4^ DCs/well) for 5 days. (A) Representative dot plots showing loss of CFSE fluorescence versus CD4 staining on day 5 for each group from one experiment. (B) The mean percentages of proliferating CD4^+^ T cells were calculated (± SEM) from five individual experiments with different donors. (C) IFN-γ levels were measured in day 5 supernatants by ELISA. Data are presented as the mean ± SEM from four individual experiments with different donors that were represented in Figure 4B. BLD = below limits of detection of the ELISA. Statistically significant differences in mean percentages and GMFI for all groups were determined by one-way ANOVA, followed by Bonferroni comparison of means (*, p<0.05; **, p<0.01; ***, p<0.001).

Enhanced maturation of human DCs by 13B47 led us to hypothesize the resulting DCs would stimulate T cells more effectively. This was tested by measuring human CD4^+^ T cell proliferation and cytokine production following co-culture with allogeneic DCs pre-treated with LVS, 13B47, or *E. coli*. T cell proliferation was measured by CFSE dilution after co-culture with infected DCs for 5 days as described previously [42], [43], [44]. An increase in the percentage of proliferating CD4^+^ T cells was observed following co-culture with 13B47-infected DCs compared to unstimulated CD4^+^ T cells (Fig. 4A and B). This increased percentage of proliferating CD4^+^ T cells was comparable to the level of proliferating T cells observed following co-culture with *E. coli*-infected DCs (Fig. 4A and B). Similar rates of CD4^+^ T cell proliferation were observed after 7 days of culture with infected DCs (data not shown). In contrast, the percentage of proliferating CD4^+^ T cells following co-culture with LVS-treated DCs was not significantly different from the baseline level of proliferation observed with unstimulated DCs (Fig. 4B).

T cell activation following co-culture with infected DCs was also assessed by cytokine production. IFN-γ concentrations in the supernatants of the DC-T cell co-cultures described above showed a similar trend to the proliferation data. CD4^+^ T cells cultured with 13B47-infected DCs produced higher levels of IFN-γ compared to those stimulated with LVS-infected DCs (Fig. 4C). T cells stimulated with bacteria alone in the absence of DCs did not proliferate or produce measurable levels of IFN-γ (data not shown). The proliferation and cytokine production data together suggest DC maturation induced by 13B47 had measurable consequences on T cells *in vitro*.

### Evaluation of the LVS FTL_0883 deletion mutant as a tularemia vaccine

**Figure 5:**
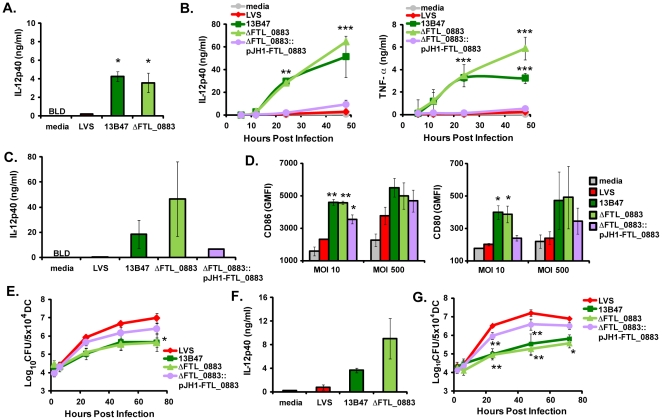
FTL_0883 deletion mutant, ΔFTL_0883, elicits maturation of DCs and is attenuated for growth similar to 13B47. Human (A–E) and murine DCs (F–G) were cultured with either LVS, 13B47, ΔFTL_0883, or ΔFTL_0883::pJH1- FTL_0883 at an MOI of 10 (A–B, D, and F) or 500 followed by gentamicin treatment (C–E, and G). For cytokines, supernatants were harvested at 24 hours (A, C, and F) or the indicated time points (B), and IL-12p40 and TNF-α were measured by ELISA. For flow cytometry experiments (D), DCs were harvested 24 hours post infection and GMFIs for CD80 and CD86 were measured. For gentamicin protection assays (E, G), DCs were infected with LVS strains at an MOI of 500 and then lysed at indicated time points to enumerate intracellular bacteria. Data are presented as the mean ± SEM from at least two independent experiments. Statistically significant differences between groups were determined by one (A, C, D, and F) or two-way ANOVA (B, E, and G), followed by Bonferroni comparison of means (*, p<0.05; **, p<0.01; ***, p<0.001). BLD = below limits of detection of the ELISA.

Protection from virulent type A *Francisella* infection is largely dependent on the development of robust T cell-mediated immunity [46]. Based on the data obtained with human cells *in vitro*, we hypothesized that vaccination with 13B47 would prolong survival and improve T cell responses compared to LVS in mice challenged with virulent *Francisella*. However, 13B47 is not optimal since it contains a transposon that could be unstable. To generate a more suitable vaccine candidate, an in-frame deletion mutant was created in LVS, ΔFTL_0883, that does not incorporate an antibiotic resistance marker [38]. Similar to human macrophages [38], more IL-12p40 and TNF-α was produced by human DCs cultured with ΔFTL_0883 than wild-type LVS (Fig. 5A–B). These cytokine levels were similar to, or greater than, that produced by DCs cultured with 13B47 (Fig. 5A–B). Moreover, IL-12p40 and TNF-α levels continued to rise from 24–48 hours when DCs were cultured with ΔFTL_0883 (Fig. 5B). To confirm the heightened stimulation of macrophages and DCs was due to deletion of FTL_0883, an in cis-complementing construct (pJH1-FTL_0883) was generated and introduced into ΔFTL_0883 [38]. Complementation of ΔFTL_0883 with the wild-type copy of the gene significantly reduced IL-12p40 and TNF-α production by human macrophages [38] and DCs (Fig. 5B). Differential induction of IL-12p40 from human DCs by FTL_0883 mutants and wild-type LVS was also observed at a higher MOI of 500 (Fig. 5C).

Changes in CD80 and CD86 expression were also evaluated in human DCs cultured with either wild-type LVS or FTL_0883 mutants. As demonstrated previously in Fig. 2, expression of CD80 and CD86 was not altered on human DCs cultured with wild-type LVS at a low MOI (Fig. 5D). Culturing of human DCs with 13B47 or ΔFTL_0883, however, caused a statistically significant increase in expression of both costimulatory molecules on the surface (Fig. 5D). CD80 and CD86 expression were also higher on DCs cultured with 13B47 or ΔFTL_0883 than on LVS-infected DCs using a higher MOI (Fig. 5D). Lower levels of CD80 and CD86 were also measured on human DCs cultured with the ΔFTL_0883 strain complemented with a wild-type copy of the gene compared to DCs cultured with ΔFTL_0883 (Fig. 5D).

Deletion of FTL_0883 in LVS reduces its ability to replicate in human and murine macrophages [38]. Additionally, the LVS mutant containing a transposon insertion in the FTL_0883 gene, 13B47, was attenuated for growth in human macrophages and replicated slowly in human DCs (Fig. 3). To measure ΔFTL_0883 replication in human DCs, DCs were infected with either wild-type LVS, 13B47, ΔFTL_0883, or the complemented strain, and lysed at various times post infection to enumerate intracellular bacteria. ΔFTL_0883 replicated more slowly in human DCs than wild-type LVS, exhibiting at least 5-fold less growth 24 hours post infection (Fig. 5E). Similar results were observed 48 and 72 hours post infection with up to a 20-fold difference in growth between ΔFTL_0883 and LVS measured 72 hours post infection (Fig. 5E). The growth kinetics for ΔFTL_0883 and 13B47 in human DCs over the 72 hour period were indistinguishable (Fig. 5E). Complementation of ΔFTL_0883 with a wild-type copy of the gene restored growth of the mutant to near wild-type levels (Fig. 5E). In summary, 13B47 and ΔFTL_0883 were similar with 1) reduced growth in human DCs, 2) increased expression of CD80 and CD86, and 3) stimulation of IL-12p40 and TNF-α production by human DCs.

To test whether the phenotypes observed in human DCs were species-specific, murine DCs were also tested with these LVS strains. IL-12p40 levels were higher in supernatants from murine DCs cultured with either 13B47 or ΔFTL_0883 compared to LVS (Fig. 5F). Growth of strains with mutations in FTL_0883 was also less than wild type in murine DCs (Fig. 5G). Similar to published work [32], LVS replicated approximately 100-fold over 24 hours in murine DCs (Fig. 5G). Both 13B47 and ΔFTL_0883 grew less robustly in murine DCs, which was less than wild-type at 24, 48, and 72 hours post infection (Fig. 5G). Growth of the ΔFTL_0883 was nearly restored to wild-type levels by the complementing construct containing a wild-type copy of the gene (Fig. 5G). These results showed that human and murine DCs responded similarly to 13B47 and ΔFTL_0883.

Having established the *in vitro* phenotypes of the ΔFTL_0883 strain, we next assessed its ability to stimulate adaptive immune responses *in vivo*. C57BL/6J mice were vaccinated by either subcutaneous (s.c.) or respiratory (i.t.) routes with LVS or ΔFTL_0883. Mice were challenged six weeks later i.t. with the type A *F. tularensis* strain Schu S4. Vaccination of C57BL/6J mice with LVS prolongs survival but does not completely protect against a secondary challenge with a type A *Francisella* strain [47], [48]. This experimental design allowed us to determine whether ΔFTL_0883 vaccination conferred better protection than LVS. All mice that received a sham vaccination with PBS succumbed to the Schu S4 infection within 5 days following challenge (Table 2). Although mice vaccinated s.c. with LVS and ΔFTL_0883 survived longer than sham-vaccinated controls, they still required euthanasia within 7 days of Schu S4 infection (Table 2). No survival differences were observed between animals vaccinated s.c. with LVS and ΔFTL_0883 (Table 2).

**Table 2:** Survival of immunized mice following intratracheal Schu S4 challenge^a^.

Route	Vaccine	Vaccination Dose	Time to Death of Individual Mice (days)	Median Time to Death (days)
Control	PBS	N/A	5, 5, 5, 5, 5	5
Subcutaneous	LVS	1×10^4^	6, 7, 7, 7, 7	7
	ΔFTL_0883	1×10^4^	6, 6, 7, 7, 7	7
Intratracheal	Experiment 1			
	LVS	1×10^3^	10,12,12, >33, >33	12^b^
	ΔFTL_0883	1×10^3^	5, 6, 6, 7, 7	6
	Experiment 2			
	LVS	2×10^3^	9, 9, 10, 11, 12	10^b^
	ΔFTL_0883	2×10^3^	6, 6, 6, 7, 7	6

a Mice were immunized with either LVS or ΔFTL_0883 at the indicated dose and then challenged with 100 CFU of Schu S4 i.t.

b Significant difference p<0.005 by log rank test.

Vaccination by a respiratory route, however, showed statistically significant differences in protective efficacy. The median time to death of mice vaccinated i.t. with LVS was approximately 10–12 days following Schu S4 challenge (Table 2). This median time to death was double the median time to death for sham-vaccinated controls (5 days, Table 2) and was similar to previous work [47]. In contrast, mice vaccinated with ΔFTL_0883 survived for a median of 6 days (Table 2). Therefore, vaccination with ΔFTL_0883 by a respiratory route provided less protection than that elicited by wild-type LVS.

**Figure 6:**
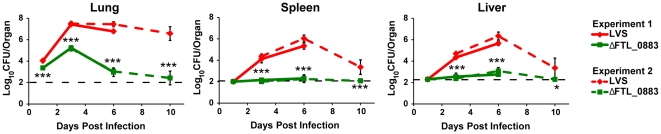
ΔFTL_0883-vaccinated mice have reduced bacterial burdens. Mice were vaccinated i.t. (1.5×10^3^ CFU) with either LVS or ΔFTL_0883 (n = 4 mice/group/time point). At indicated time points (days 1, 3, and 6 for experiment 1; days 3, 6, and 10 for experiment 2), mice were sacrificed and the CFU/organ was determined as described in Materials and Methods. The limits of detection for the lung, spleen, and liver are depicted by the black dashed lines and were 100, 100, and 200 CFU, respectively. Data are presented as mean ± SD for each individual experiment. Statistically significant differences between groups in each experiment were determined by two-way ANOVA, followed by Bonferroni comparison of means (*, p<0.05; ***, p<0.001).

To investigate the differences in the protection elicited by the two strains, we evaluated bacterial burdens in the lung and peripheral organs following respiratory vaccination. LVS replicated exponentially in the lung for the first three days following i.t. immunization (Fig. 6). The lung bacterial burden remained steady until day 6 post immunization and then slowly began to decline up to day 10 (Fig. 6). Dissemination to the spleen and liver occurred at day 3 with LVS burden peaking at day 6 and being cleared by day 10 (Fig. 6). Despite comparable doses of bacteria used in the vaccinations, lower levels of ΔFTL_0883 were detected at all time points in the lung and beginning at day 3 in peripheral organs post immunization (Fig. 6). While clearance of LVS from the lung does not occur until 22 days post infection [49], ΔFTL_0883 was cleared more rapidly at approximately 10 days post infection (Fig. 6). Viable ΔFTL_0883 were measured in the spleens and livers of seven of eight mice by day 6, but none were detected in these organs at day 10 (Fig. 6). Therefore, LVS achieved higher numbers for a longer period of time in the lung and periphery following vaccination.

**Figure 7:**
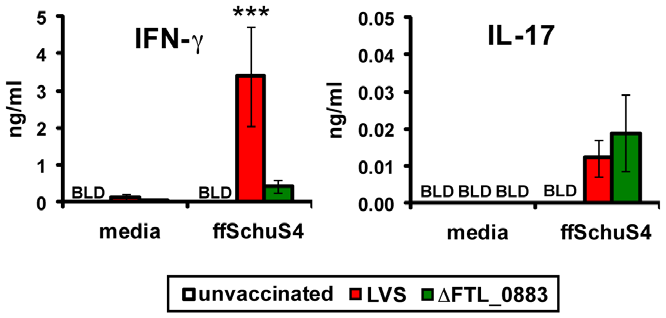
Cells from mice vaccinated with LVS produce more IFN-γ after re-stimulation. Mice were vaccinated i.t. (8×10^3^ CFU) with either LVS (n = 3) or ΔFTL_0883 (n = 4). Age-matched naïve mice (n = 3) served as negative controls. Lung cells were isolated from vaccinated and unvaccinated mice 30 days post vaccination and incubated with formalin-fixed SchuS4 (ffSchu S4) and BMDCs. Culture supernatants were collected 48 hours later and assessed for multiple cytokines and chemokines using the Milliplex 32-plex Mouse Cytokine/Chemokine Panel (Millipore) on a Bio-Plex system (Bio-Rad Laboratories, Inc.). Baseline levels of cytokine/chemokine production were established by the culturing of lung cells in media alone (no antigen). Data are presented as the mean ± SD of triplicate wells from one individual experiment. Another experiment of similar design was performed with a vaccination dose of 500 CFU i.t., and IL-17 and IFN-γ levels were measured by ELISA. Similar results were observed in both experiments. Statistically significant differences in cytokine/chemokine production were determined by a one-way ANOVA, followed by Bonferroni comparison of means. (**, p<0.01; ***, p<0.001 for LVS v. ΔFTL_0883 following ffSchu S4 re-stimulation).

We next sought an immunological explanation for the performance of ΔFTL_0883 vaccination. We hypothesized wild-type LVS induced superior T cell responses than ΔFTL_0883, and measured cytokine and chemokine responses by lung cells after i.t. vaccination. Cells were harvested from the lungs of LVS- and ΔFTL_0883-vaccinated mice and were re-stimulated *in vitro* with ffSchu S4. Cells from mice vaccinated with LVS produced higher amounts of IFN-γ with re-stimulation than cells from naïve mice or those that received ΔFTL_0883 (Fig. 7). IFN-γ production by lung cells from mice vaccinated with ΔFTL_0883, however, was not statistically significantly different than naïve controls (Fig. 7). Increasing the vaccination dose of ΔFTL_0883 by three-fold failed to improve IFN-γ responses by the lung cells (data not shown). Consistent with the IFN-γ results, the IFN-γ inducible chemokine MIG was also higher in cultures from mice vaccinated with LVS (data not shown). In contrast to IFN-γ, cells from both vaccination groups produced comparable amounts of IL-17 after re-stimulation. A 2–3 fold increase in IL-17 production was observed in lung cells from mice vaccinated with ΔFTL_0883 and LVS compared to naïve controls (Fig. 7). No other statistically significant differences were consistently detected in the other cytokines and chemokines that were tested (data not shown). Therefore, the protection elicited by LVS against Schu S4 challenge correlated with IFN-γ production by lung cells after re-stimulation with antigen.

## Discussion

Several studies, including this one, indicate LVS poorly stimulates innate immune cells [24], [25], [27], [29], [30], [32]. This suggests insufficient activation of DCs could contribute to incomplete protection engendered by LVS. In this study, we sought to improve vaccine efficacy with a LVS strain that stimulated APCs better than wild-type LVS. The LVS mutants used in this study (Table 1) were selected based on specific characteristics. All three of these genes (FTL_1415, FTL_1664, and FTL_0883) have been identified in negative selection screens in *F. novicida* and/or LVS to be necessary for growth and/or survival in mice [50], [51]. The ΔcapC mutant was of interest because another LVS mutant in the capBCA operon, ΔcapB, afforded protection in BALB/c mice against challenge with the virulent *Francisella* strain Schu S4 [49]. Mutation of FTL_1664 in LVS resulted in diminished uptake by human DCs [34], which may impact DC activation. Recently, our laboratory has shown that LVS FTL_0883 mutants like 13B47 stimulate innate immune cells and are attenuated *in vitro* and *in vivo*, making this mutant a possible vaccine candidate [38].

Strains with mutations in the FTL_0883 locus of LVS showed promise based on *in vitro* results. The 13B47 and ΔFTL_0883 derivatives of LVS stimulated human DCs and macrophages (Fig. 1, 2, and 5), which was associated with better stimulation of T cells *in vitro* (Fig. 4). Contrary to our hypothesis, however, improving APC stimulation *in vitro* with the ΔFTL_0883 strain did not enhance protection *in vivo*. The median time to death doubled in mice vaccinated in the respiratory tract with LVS compared to naïve animals. In contrast, the median time to death of mice vaccinated with ΔFTL_0883 was similar to naïve animals (Table 2). Enhancing DC stimulation with ΔFTL_0883, therefore, failed to establish a protective immune response.

The poor performance of ΔFTL_0883 as a vaccine may be due directly to its attenuation. Mutation of the FTL_0883 locus in LVS attenuates growth in macrophages and DCs (Fig. 3, 5E, and 5G, and [38]). In addition, bacterial burdens in the lung and periphery of ΔFTL_0883-vaccinated mice are less than in animals receiving wild type (Fig. 6). Based on these findings, the attenuation and accelerated clearance of ΔFTL_0883 *in vivo* may prevent a sufficient adaptive immune response from being established. Consistent with this model, restimulation of lung cells isolated from ΔFTL_0883-vaccinated mice produced less IFN-γ than mice receiving wild type (Fig. 7). Since IFN-γ is a critical mediator of protective immunity against tularemia [48], [52], [53], the diminished IFN-γ response we observed following restimulation likely contributed to the lack of protection after vaccination with ΔFTL_0883.

Additional factors may also contribute to the vaccination results seen in this study. Though the molecular function of the protein encoded by FTL_0883 is unknown, it is possible that protective antigens may not be expressed since spermine responsiveness and transcription are altered after mutation of FTL_0883 [38]. Alternatively, different cytokine profiles stimulated in the host by the ΔFTL_0883 mutant may influence the vaccine performance. Inflammatory signals such as IL-12 can modulate T cell differentiation, promoting the generation of more short-lived effector cells compared to memory precursors [54]. The higher levels of IL-12 stimulated by ΔFTL_0883 (Fig. 5) may have shifted T cell development, impairing the development of memory cells. Each of these possibilities is consistent with the reduced IFN-γ observed during re-stimulation of lung cells with antigen *in vitro*. The mechanism(s) accounting for the poor recall responses observed with ΔFTL_0883 is currently being investigated.

The results presented here with the FTL_0883 mutants share common outcomes with studies of other genetically altered *Francisella*. Mutation of *iglC* or *mglA*, genes important for intracellular growth of *Francisella*, or deletion of the *purMCD* purine biosynthesis operon resulted in highly attenuated strains that did not provide better protection than LVS against virulent *Francisella* challenge [55], [56], [57]. In contrast, vaccination with a Δ*clpB* mutant in the Schu S4 background is superior to wild-type LVS [21]. A greater IFN-γ response was measured four days after challenge of mice vaccinated with the more successful Δ*clpB* mutant than those vaccinated with LVS [58]. Coupled with our results, IFN-γ responses measured during restimulation could be a useful predictor of vaccine efficacy.

Several recent studies have shown that IL-17 is also required for control of *F. tularensis* growth and the generation of an effective Th1 response following pulmonary challenge [59], [60], [61]. Although the role of IL-17 in the immune response to acute *F. tularensis* infection has been characterized [59], [60], [61], its role in vaccination against tularemia remains to be elucidated. Paranavitana *et al.* demonstrated that PBMCs from LVS-vaccinated individuals produce high levels of IL-17 following *in vitro* re-stimulation [62]. Similarly, we have shown that pulmonary vaccination of mice with LVS results in an increase in IL-17 compared to naïve controls (Fig. 7). Production of IL-17, however, did not correlate with vaccine efficacy since comparable levels of IL-17 were produced by cells from mice receiving wild-type or ΔFTL_0883 vaccinations (Fig. 7). Additionally, neutralization of IL-17 in mice successfully protected by a Schu S4 Δ*clpB* vaccine did not reduce survival after a pulmonary type A challenge despite increasing bacterial burden [58]. Therefore, IL-17 alone is not sufficient to predict vaccine efficacy.

Defining an optimal strategy for vaccine development remains a significant challenge for many pathogens. Improving APC stimulation using genetic modifications of LVS in this project failed to improve protection against a virulent *F. tularensis* strain. In addition, modeling vaccination and challenge *in vitro* with human cells did not predict *in vitro* responses in mice. Comparison of different vaccine strains and the protection conferred, however, confirmed IFN-γ production as a potential correlate of protection. A similar experimental approach by Shen *et al.*. successfully characterized the immune response to *Francisella* strains that varied in vaccine efficacy [58]. Nevertheless, our current study and that of Shen *et al.* are limited by the conditions tested (the number of vaccine and mouse strains used), the limited number of output variables measured (relying primarily on multiplex cytokine measurements), and the timing of sampling (responses tested after challenge *in vitro* or *in vivo*). This leaves open the possibility that more comprehensive investigations could yield additional insights. Recently, a systems-wide analysis of vaccine responses against yellow fever has met with significant success [63], [64]. In this approach, genome-wide transcriptional studies using microarrays provided a broader assessment of in vivo host responses to vaccination [63]. A seminal application of these concepts to Francisella was also recently published by DePascalis et al. [65]. Here, an in vitro lymphocyte-macrophage co-culture was used to model the immune responses elicited by LVS vaccines of varying efficacies [65]. Analysis of 84 immunologically-relevant genes by real time PCR identified a list of immune mediators whose expression pattern correlated with protection from F. tularensis infection, including IFN-g [65]. These higher order analyses, which integrate multi-parameter data sets of a variety of measurements, combined with traditional testing of specific hypotheses will continue to yield insights into correlates of protection and biological response modifiers that may be exploited during acute infection and vaccination.
